# The Effect of Technology-Based Home Cardiac Rehabilitation on Risk Factor Modifications in Coronary Heart Disease Patients. A Systematic Review and Meta-Analysis

**DOI:** 10.31083/j.rcm2502059

**Published:** 2024-02-05

**Authors:** Yemei Hu, Kun Ding, Gang Wu, Xuedong Li, Jun Li, Zhuo Shang

**Affiliations:** ^1^Department of Cardiology, Bengbu Second People’s Hospital, 233000 Bengbu, Anhui, China

**Keywords:** cardiac rehabilitation, coronary heart disease, second prevention, lifestyle change, monitoring devices

## Abstract

**Background:**

The delivery channels and approaches related to cardiac rehabilitation (CR), such as eHealth, mHealth, and telehealth, are evolving. Several studies have identified their effects on patients with coronary heart disease, although no studies have focused on all the approaches collectively.

**Methods:**

Randomized controlled trials have investigated lipid profiles, through systolic blood pressure (SBP), diastolic blood pressure (DBP), and body mass index (BMI). Stata software was used for analysis, while Egger’s linear regression test and Begg’s funnel plot were also applied.

**Results:**

Technology-based home CR revealed significantly lower total cholesterol (TC) levels (standardized mean difference (SMD) = –0.19; 95% confidence interval [CI]: [–0.27, –0.11]); triglyceride (TG) levels (SMD = –0.26; 95% CI: [–0.35, 0.17]); low-density lipoprotein (LDL) levels (SMD = –0.18; 95% CI: [ –0.25, –0.11]); SBP (SMD = –0.26; 95% CI: [–0.33, –0.19]); DBP (SMD = –0.24; 95% CI: [–0.32, –0.16]); BMI (SMD = –0.12; 95% CI: [–0.18, –0.05]), and improved high-density lipoprotein (HDL) levels (SMD = 0.22; 95% CI: [0.14, 0.31]).

**Conclusions:**

Technology-based home CR can be used to lower TC, TG, and LDL levels, alongside the BMI, SBP, and DBP indexes, while also raising HDL levels; thus, its use should be widely promoted.

## 1. Introduction

Coronary heart disease (CHD) is a major global cause of death and morbidity [1], which represents a huge public health concern as well as a large economic burden because patients with CHD are at serious risk of having a myocardial infarction, requiring hospital readmission, and even dying prematurely [2, 3]. International guidelines have strongly recommended undertaking cardiac rehabilitation (CR), evidence-based pharmacological therapy, optimization of cardiovascular risk factors, and adherence to diet and physical activity to relieve the risks associated with CHD [3, 4].

Compared with center-based CR, there are several advantages to technology-based home CR: (1) It allows patients to access cardiac rehabilitation programs from their homes, eliminating the need to travel to a healthcare facility, which can be especially beneficial for individuals who live in remote areas. (2) Digital platforms can collect and analyze data from wearable devices to customize exercise regimens and design treatment plans specifically to the needs of individual patients. (3) Real-time data on a patient’s heart rate, blood pressure, and exercise efficiency can be obtained using wearable technology and apps. In order to improve patient safety, health providers can remotely monitor this data and take appropriate action.

With the advancement in technology, CR delivery channels and approaches have emerged, such as eHealth, mHealth, and Telehealth. Subsequently, throughout this paper, “technology-based interventions” refers to patient care activities that involve the use of the internet, digital/mobile appliances, and telephones, such as evaluations, monitoring, training, and encouragement of a healthy lifestyle. Previous studies concentrated on a single form of CR delivery channels and approaches in patients with CHD, meaning there is no research on technology-based CR delivery, which comprises all approaches related to technology. In addition, according to the currently available evidence, CR makes secondary prevention possible, which is essential after diagnosis because managing risk factors is a crucial step in the management of recurrence [5, 6, 7], whereby controlling cholesterol levels and blood pressure are crucial secondary prevention goals. According to the meta-analysis by Ettehad *et al*. [8], which analyzed data from more than 600,000 adults, a 10 mmHg decline in systolic blood pressure (SBP) is linked to a 20% decline in major cardiovascular events and a 13% decrease in all-cause death. Additionally, there is a 21% decrease in cardiovascular events for every 1 mmol/L decrease in low-density lipoprotein (LDL) cholesterol levels [8]. In a previous study, Chong *et al*. [9] analyzed the efficacy of technology-assisted CR in CHD patients; however, they only analyzed SBP, total cholesterol (TC), and circumference, meaning the evidence for reducing risk factors was insufficient. Meanwhile, considering the importance of reducing cardiovascular risk factors in patients with CHD, we performed a meta-analysis to summarize and update all technology-associated approaches in home CR and determine the efficacy of using technology-based home cardiac rehabilitation to control risk variables in patients with CHD.

## 2. Material and Methods

This systematic review and meta-analysis were performed to investigate the effect of home-based CR in patients with CHD in accordance with the Preferred Reporting Items for Systematic Reviews and Meta-Analyses (PRISMA) guidelines.

### 2.1 Literature Search Strategy

MEDLINE, PubMed, EMBASE (Ovid), Web of Science, Scopus, and Cochrane Central Register of Controlled Trials were thoroughly searched to identify any relevant studies that had been published up to June 30, 2022. Based on Medical Subject Headings (MeSH), we conducted a search using phrases such as “cardiac rehabilitation” or “lifestyle modification” or “physical training” or “preventative strategy” alongside “CHD” or “CAD (coronary artery disease)” or “AMI (acute myocardial infarction)” or “ACS (acute coronary syndrome)” and “lipid profile” or “LDL” or “high-density lipoprotein (HDL)” or “triglyceride (TG)” or “total cholesterol (TC)”, and risk factors, such as “body mass index (BMI)”, “systolic blood pressure (SBP)”, and “diastolic blood pressure (DBP)”. The reference lists of the articles were manually screened for potentially eligible studies, alongside the reference lists of previously published clinical trials and conference abstracts from the American Heart Association (AHA), and European Society of Cardiology (ESC) scientific sessions.

### 2.2 Inclusion and Exclusion Criteria for Study Collections

Studies that met the following standards were included: (1) home-based cardiac rehabilitation-controlled trials, regardless of allocation concealment or blinding; (2) included patients over 18 years of age with CHD; (3) contained patients undergoing home-based CR in the intervention group; used patients that received usual care or medication in the control group; (4) applied measuring markers as risk factors, such as lipid profiles (HDL, LDL, TC, TG, BMI, and blood pressure). Studies were excluded if they: (1) were not in English; (2) had incomplete information or data; (3) contained center-based or exercise-based CR control groups.

### 2.3 Data Extraction

Two researchers (YH and JL) independently reviewed each study, and a third researcher (KD) settled any disagreements. From the listed studies, researchers independently retrieved the data (title, author information, publishing year, and study origin), as well as demographic characteristics (gender, age, nation, and sample size) for both the treatment and control groups and obtained measured outcomes. A consensus was reached following the occurrence of any contradictions.

### 2.4 Quality Assessment 

We evaluated the quality of the studies using the Cochrane risk of bias tool [10]. The instrument evaluates the quality of a study with regard to selection bias, performance bias, detection bias, attrition bias, reporting bias, and intention-to-treat analysis. A third researcher assessed any discrepancies after two reviewers separately assessed the quality of the studies.

### 2.5 Statistical Analysis

Stata statistical software (version 13.0; Stata Corp., College Station, TX, USA) was used to conduct statistical analyses. A standardized mean difference (SMD) and 95% confidence interval (CI) were used to assess the effect size. The Q test and $I^{2}$ analysis were employed to measure study heterogeneity. The source of heterogeneity was identified using meta-subgroup analysis. Publication bias was analyzed by Egger’s linear regression test and Begg’s funnel plot [11]. The statistical significance level was set at *p* < 0.05.

## 3. Results

### 3.1 Literature Selection

**Fig. 1.**
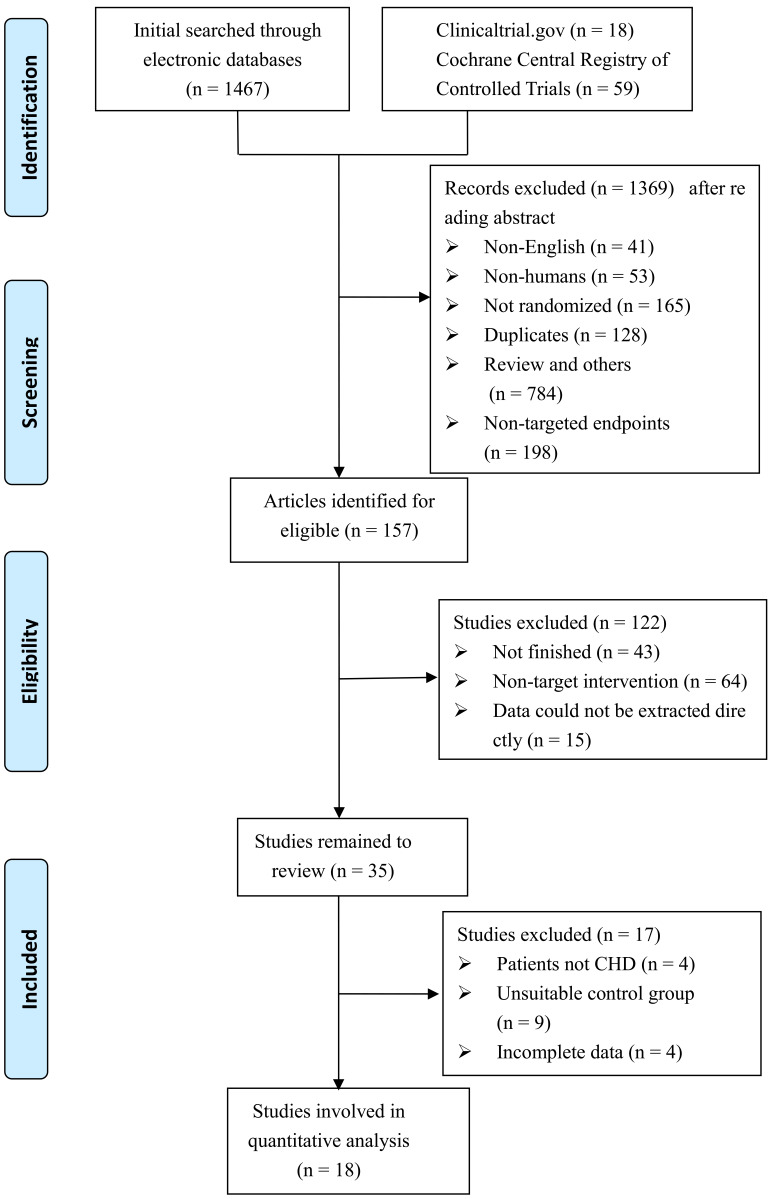
**Diagram of study selection procedures**. CHD, coronary heart disease.

In total, we found 1526 potentially relevant articles across all resources: PubMed (859), Cochrane (59), Embase (590), and clinical trials (18). An electronic search yielded a total of 1526 potentially relevant articles. A total of 1369 publications were removed after careful examination of their titles and abstracts, of which 784 were reviews and other forms of references, 41 were written in languages other than English, 53 were research studies on non-human species, 128 were duplicates, and 198 contained untargeted outcome measures. Specific criteria were used to analyze the 157 papers that remained. Following a review of the full text, 122 studies were eliminated as they were either incomplete (n = 43), involved non-target therapies (n = 64), contained information that could not be abstracted properly (n = 15), had non-CHD patients (n = 4), included inappropriate comparisons (n = 9), or had missing information (n = 4) (Fig. 1).

### 3.2 Characteristics of Included Studies

Eventually, 18 papers were considered in the meta-analysis [12, 13, 14, 15, 16, 17, 18, 19, 20, 21, 22, 23, 24, 25, 26, 27, 28, 29]. All the included investigations, which comprised 3661 patients, were published between 2007 and 2022. There were between 15 and 822 patients in the sample selection (median, 131). The age range of all included patients was 51.7–77.3 years. Twenty studies used a two-arm parallel design, while only one study used a three-arm parallel design (home-based CR and comparison). Patients in the study by Avila *et al*. [22] were divided into three groups (home-based, center-based, and control groups). Five studies were performed in Australia, two in Canada, two in Belgium, one in Sweden, one in China, one in Spain, one in New Zealand, one in the Netherlands, and one collaborative study in Australia with cooperation from New Zealand. The collected studies used a variety of endpoints, with 14 studies collecting the TC measurements as the endpoint, 13 studies using HDL levels, 15 trials using LDL levels, 11 studies considering the TG level, 17 studies applied SBP as the endpoint, 15 studies used DBP, and 16 studies used BMI (Table 1, Ref. [12, 13, 14, 15, 16, 17, 18, 19, 20, 21, 22, 23, 24, 25, 26, 27, 28, 29]).

**Table 1.** **Characteristics of all included trials**.

Author	Disease type	Country	No. of patients		Mean age (SD)		Gender (M/F)		Outcome measures	Follow-up	Interventions
			C	T	C	T	C	T			
Avila *et al*. 2018 [22]	CAD	Belgium	26	28	61.7 (7.7)	58.6 (13)	3/23	4/24	SBP, DBP, BMI	12 weeks	Received training for the first three sessions under the supervision of the investigator; received an individualized aerobic exercise prescription recommending at least 150 min of exercise per week (preferably 6–7 days/week) at an individually determined target heart rate, corresponding to moderate intensity in their home environment during the 12-week intervention
Chow *et al*. 2015 [16]	CHD	Australia	352	358	57.9 (9.1)	57.3 (9.3)	287/65	295/63	TC, TG, HDL, LDL, SBP, DBP, BMI	6 months	Text messages provided advice, motivation, reminders, and support to change lifestyle behaviors
Dalli Peydró *et al*. 2022 [29]	ACS	Spain	31	28	57.5 (9.0)	54.7 (9.9)	27/4	27/1	TC SBP, DBP	10 months	Four supervised sessions of exercise were completed. Physical activity consisted of walking down a corridor and adjusting their pace to attain a target heart rate as measured by their smartphone and heart rate monitor
Dorje *et al*. 2019 [23]	MI and unstable or stable angina	Australia	156	156	59.1(9.4)	61.9 (8.7)	128/28	126/30	TC, TG, HDL, LDL, SBP, DBP, BMI	4 months	SMART-CR/SP system employed WeChat’s powerful functions in social media communication, online education, physical activity tracking, blood pressure, and heart-rate monitoring (linked to an external monitor), wireless data transfer, and multimedia—to support the delivery of a comprehensive home cardiac rehabilitation and secondary prevention program
Frederix *et al*. 2015 [17]	CAD	Belgium	70	69	61 (8)	61 (9)	55/15	59/10	SBP, DBP, BMI	24 weeks	Semi-automatic telecoaching system to provide the patients with feedback via email and short message service (SMS) messaging (once a week), encouraging them to gradually achieve predefined exercise training goals; specific patient-related advice on dietary requirements and smoking cessation was also provided as part of the telecoaching
Johnston *et al*. 2016 [20]	MI	Sweden	86	80	56.8 (8.0)	58.4 (8.6)	71/15	63/17	LDL	6 months	Received a complete interactive patient support tool (Web-based application) installed on their own smartphones containing an extended drug adherence e-diary and secondary prevention educational modules
Lear *et al*. 2014 [14]	CVD	Canada	38	40	59.2 (10.7)	58.6 (9)	34/4	32/8	TC, TG, HDL, LDL, SBP, DBP, BMI	16 months	Online intake forms (medical, risk factor, and lifestyle), scheduled one-on-one chat sessions with the program nurse case manager, exercise specialist, and dietitian (3 times each during the 16 weeks), alongside weekly education sessions
Maddison *et al*. 2019 [24]	CHD	Australia and New Zealand	82	80	61 (13.2)	61.5 (12.2)	69/13	70/10	TC, TG, HDL, LDL, SBP, DBP, BMI	24 weeks	REMOTE-CR comprised 12 weeks of individualized exercise prescription, exercise monitoring, and coaching plus theory-based behavioral strategies to promote exercise and habitual lifestyle alterations, delivered via a bespoke telerehabilitation platform
Pfaeffli *et al*. 2015 [18]	CHD	New Zealand	62	61	59.9 (11.8)	59.0 (10.5)	52/10	48/13	TC, HDL, LDL, SBP, DBP, BMI	6 months	A 24-week mHealth program sent by automated daily text messages and access to a supporting website commencing within a week of the baseline assessment
Santo *et al*. 2019 [25]	CHD	Australia	107	56	58.4 (9.0)	56.8 (8.6)	93/14	50/6	TC, LDL, SBP, DBP	3 months	Basic app provided simple daily reminders, similar to an alarm or text message, to prompt the participants to take their medications at the correct time every day
Uddin *et al*. 2020 [27]	CHD	USA	71	71	54 (6)	55 (6)	66/5	63/8	TC, TG, HDL, LDL, SBP, DBP, BMI	12 months	Calls were made by qualified physiotherapists, trained by the research team regarding the CR advice booklet and exercise program. The physiotherapists answered any patient questions and reminded them to follow the CR advice booklet and exercise program and to attend their next hospital appointment
Varnfield *et al*. 2014 [15]	Post-MI	Australia	41	53	56.2 (10.1)	54.9 (9.6)	34/7	48/5	TC, TG, HDL, LDL, SBP, DBP	6 months	Health and exercise monitoring, motivational and educational material delivery, and weekly mentoring consultations. CAP-CR uptake completion rates compared with TCR using intention-to-treat analyses
Vernooij *et al*. 2012 [13]	CAD	Netherland	164	166	60.7 (7.8)	59.2 (8.9)	128/36	118/48	TC, TG, HDL, LDL, SBP, DBP, BMI	12 months	Personalized website; mail communication via the website with a nurse practitioner; monitoring of disease control, and drug treatment
Widmer *et al*. 2015 [19]	CVD	USA	19	25	70.4 (9.9)	60.2 (12.1)	17/2	19/6	TC, TG, HDL, LDL, SBP, DBP, BMI	3 months	Used online platforms to monitor their own CVD indices, diet, and exercise; adherence, and were tasked with accessing educational materials in a personal health assistant
Widmer *et al*. 2017 [21]	ACS PCI	USA	40	40	63.6 (10.9)	62.5 (10.7)	29/5	29/8	TC, TG, HDL, LDL, SBP, DBP, BMI	180 days	Online and smartphone-based CR platform, which requested patients to report their dietary and exercise habits throughout CR as well as educational information toward the patients’ healthy lifestyles
Yudi *et al*. 2021 [28]	ACS	Australia	83	85	56.8 (9.9)	56.2 (10.2)	71/12	69/16	TC, TG, HDL, LDL, SBP, DBP, BMI	8 weeks	Exercise prescription; dynamic tracking of cardiovascular risk factors; assessment of dietary habits; health education; education on secondary prevention pharmacotherapy as well as interactive and personalized feedback and support
Zheng *et al*. 2019 [26]	AMI	China	411	411	56.2 (9.3)	56.6 (9.7)	353/58	353/58	LDL, SBP, BMI	6 months	The messages provided educational and motivational information related to disease-specific knowledge, risk-factor control, physical activity, and medication adherence
Zutz *et al*. 2007 [12]	CAD	Canada	8	7	58 (4)	59 (12)	7/1	5/2	TC, TG, HDL, LDL, SBP, DBP, BMI	12 weeks	Online intake forms, one-on-one chat sessions with a nurse, dietitian, and exercise specialist

ACS, acute coronary syndrome; AMI, acute myocardial infarction; BMI, body mass index; CAD, coronary artery disease; CHD, coronary heart disease; CVD, coronary vascular disease; DBP, diastolic blood pressure; HDL, high-density lipoprotein; LDL, low-density lipoprotein; MI, myocardial infarction; PCI, percutaneous coronary intervention; SBP, systolic blood pressure; TC, total cholesterol; TG, triglycerides; M, male; F, female; SMART-CR/SP, smart-cardiac rehabilitation, and secondary prevention; REMOTE-CR, remote cardiac rehabilitation; CAP-CR, care assessment platform cardiac rehabilitation; TCR, traditional center-based cardiac rehabilitation; CR, cardiac rehabilitation; C, control group; T, treatment group.

### 3.3 Meta-Analysis Results

#### 3.3.1 TC Levels

**Fig. 2.**
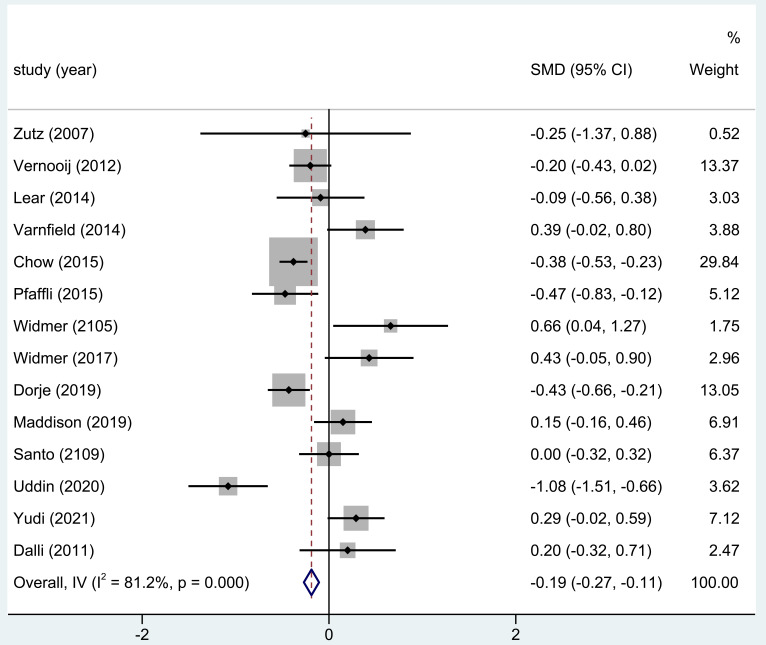
**The effect of home CR on TC levels**. TC, total cholesterol; CR, cardiac rehabilitation; SMD, standardized mean difference; CI, confidence interval.

A total of 14 of the 18 studies contained in this analysis indicated TC levels post-home-based CR. Meaningful differences in TC serum levels were observed between CR and the compared arms (standardized mean difference (SMD) = –0.19; 95% CI: [–0.27, –0.11]; *p* < 0.001, Fig. 2).

#### 3.3.2 HDL Levels

**Fig. 3.**
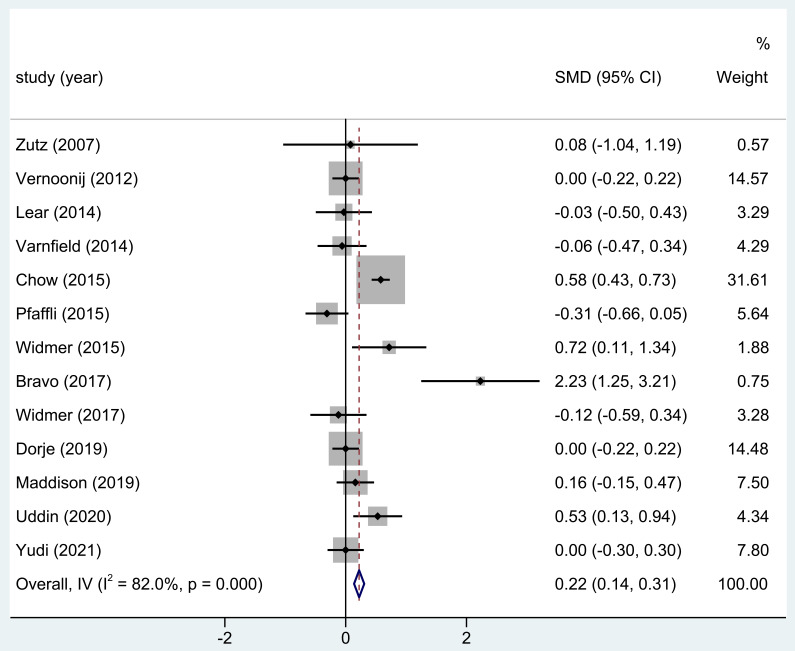
**The effect of home CR treatment on HDL levels**. CR, cardiac rehabilitation; HDL, high-density lipoprotein; SMD, standardized mean difference; CI, confidence interval.

Thirteen of the eighteen studies considered in this analysis used HDL levels as the outcome metric following home-based CR. The outcomes demonstrated that in four investigations, the CR group’s HDL serum levels were noticeably greater than those of the control group. The results of the entire research exhibited notable differences in HDL levels post-home-based CR (SMD = 0.22; 95% CI: [0.14, 0.31]; *p* < 0.001, Fig. 3).

#### 3.3.3 LDL levels

**Fig. 4.**
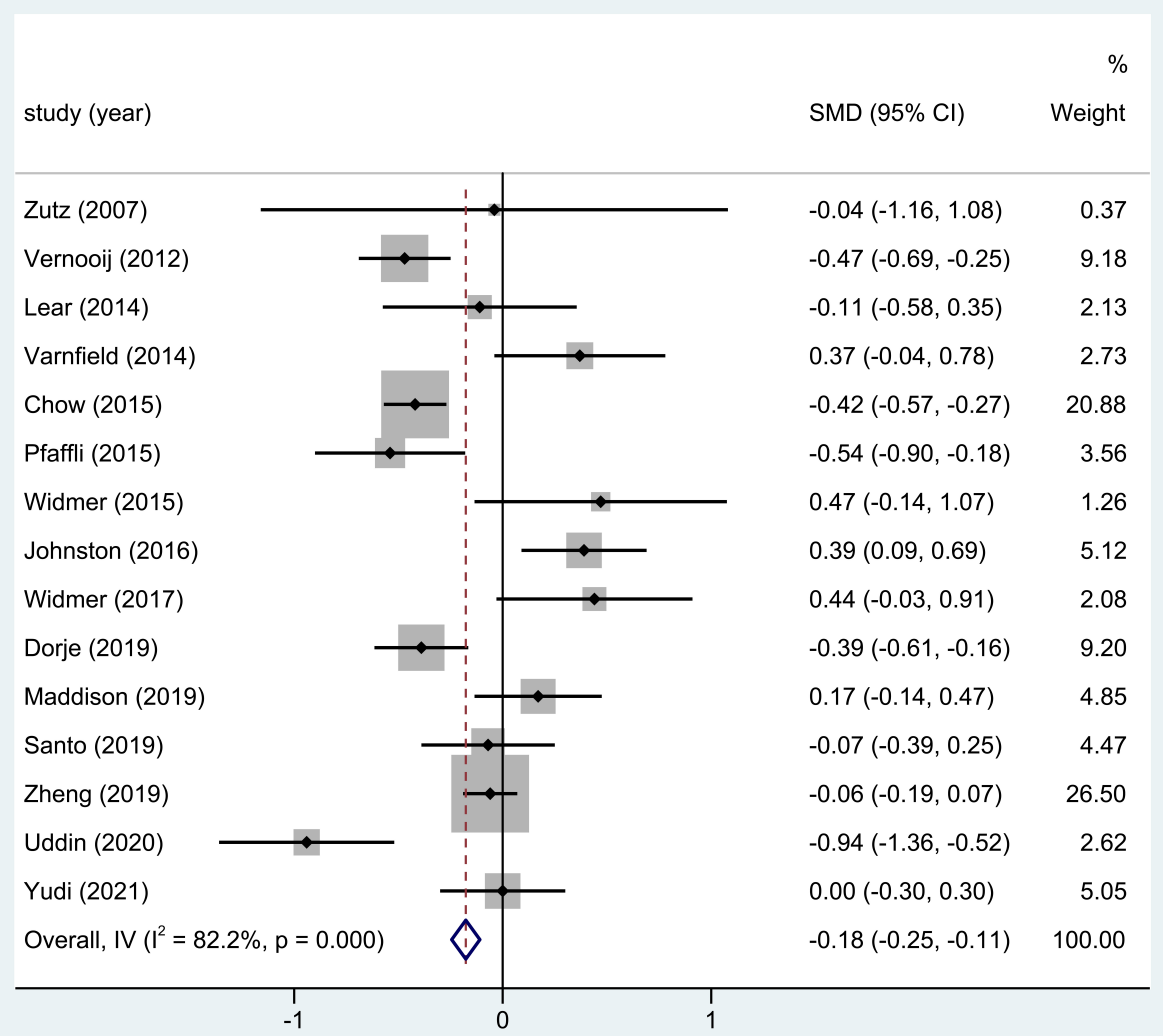
**The effect of home CR treatment on LDL levels**. CR, cardiac rehabilitation; LDL, low-density lipoprotein; SMD, standardized mean difference; CI, confidence interval.

A total of 15 out of the 18 studies involved in this research noted the LDL levels following home-based CR. The result displayed that in three investigations, the blood LDL levels in the CR group were substantially lower than in the control group. Serum LDL levels after home-based CR significantly differed in the study’s overall findings (SMD = –0.18; 95% CI: [–0.25, –0.11]; *p* = 0, Fig. 4).

#### 3.3.4 TG Levels

**Fig. 5.**
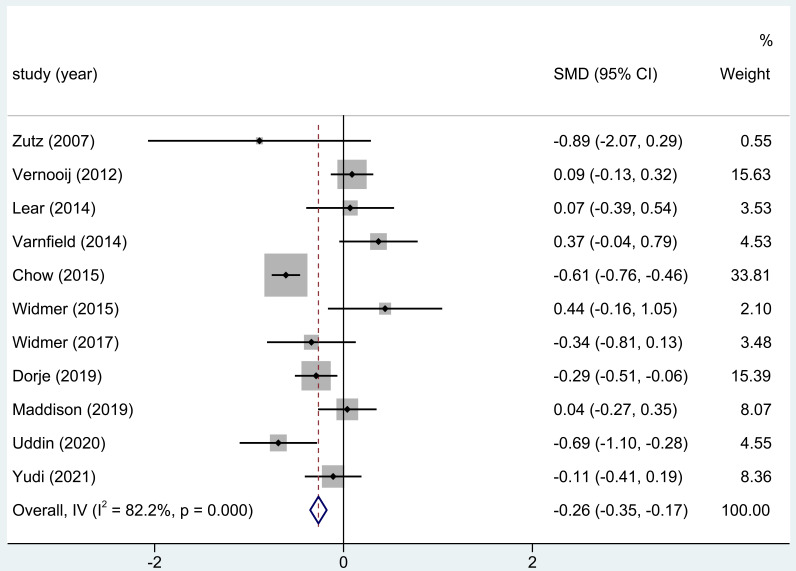
**The effect of home CR treatment on TG levels**. CR, cardiac rehabilitation; TG, triglyceride; SMD, standardized mean difference; CI, confidence interval.

TG levels were recorded after home-based CR in 11 of the 18 trials comprised in this analysis. The whole study results demonstrated substantial variations in the TG levels (SMD = –0.26; 95% CI: [–0.35, –0.17]) following home-based CR. Additionally, 17 of the 18 trials involved in this meta-analysis reported SBP levels post-home-based CR. The outcomes of the entire study showed notable differences in the SBP after home-based CR (SMD = –0.26; 95% CI: [–0.33, –0.19], Fig. 5).

#### 3.3.5 SBP Levels

**Fig. 6.**
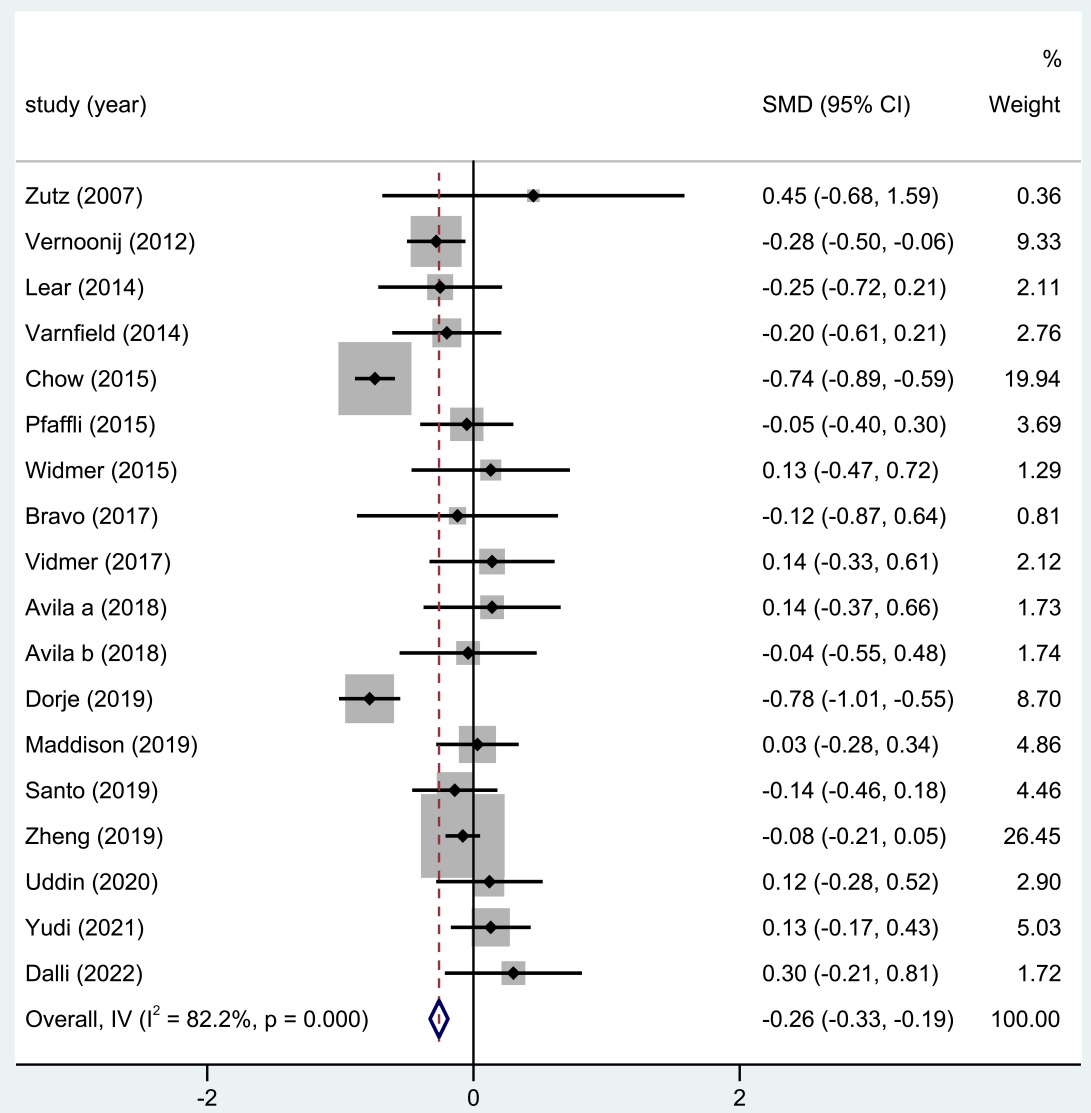
**The effect of home CR treatment on SBP**. CR, cardiac rehabilitation; SBP, systolic blood pressure; SMD, standardized mean difference; CI, confidence interval.

SBP levels were reported in 17 of the trials included in this analysis. The outcome of the entire study revealed substantially significant differences in the SBP after home-based CR (SMD = –0.26; 95% CI: [–0.33, –0.19], Fig. 6).

#### 3.3.6 DBP Levels

**Fig. 7.**
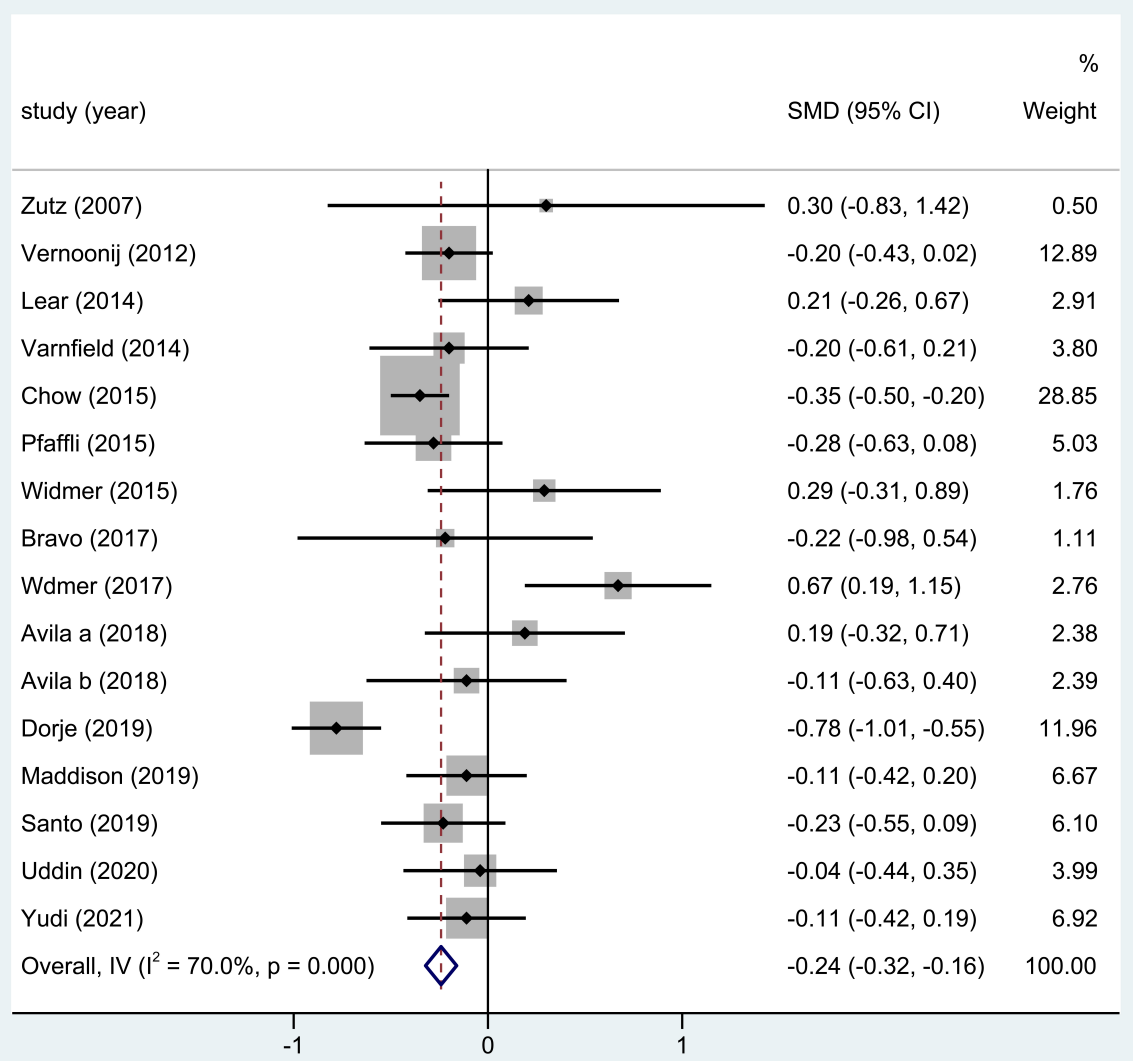
**The effect of home CR treatment on DBP**. CR, cardiac rehabilitation; DBP, diastolic blood pressure; SMD, standardized mean difference; CI, confidence interval.

DBP levels were reported in 15 of the trials included in this analysis. The outcome of the entire study revealed substantially significant differences in the DBP after home-based CR (SMD = –0.24; 95% CI: [–0.32, –0.16], Fig. 7).

#### 3.3.7 BMI Levels

**Fig. 8.**
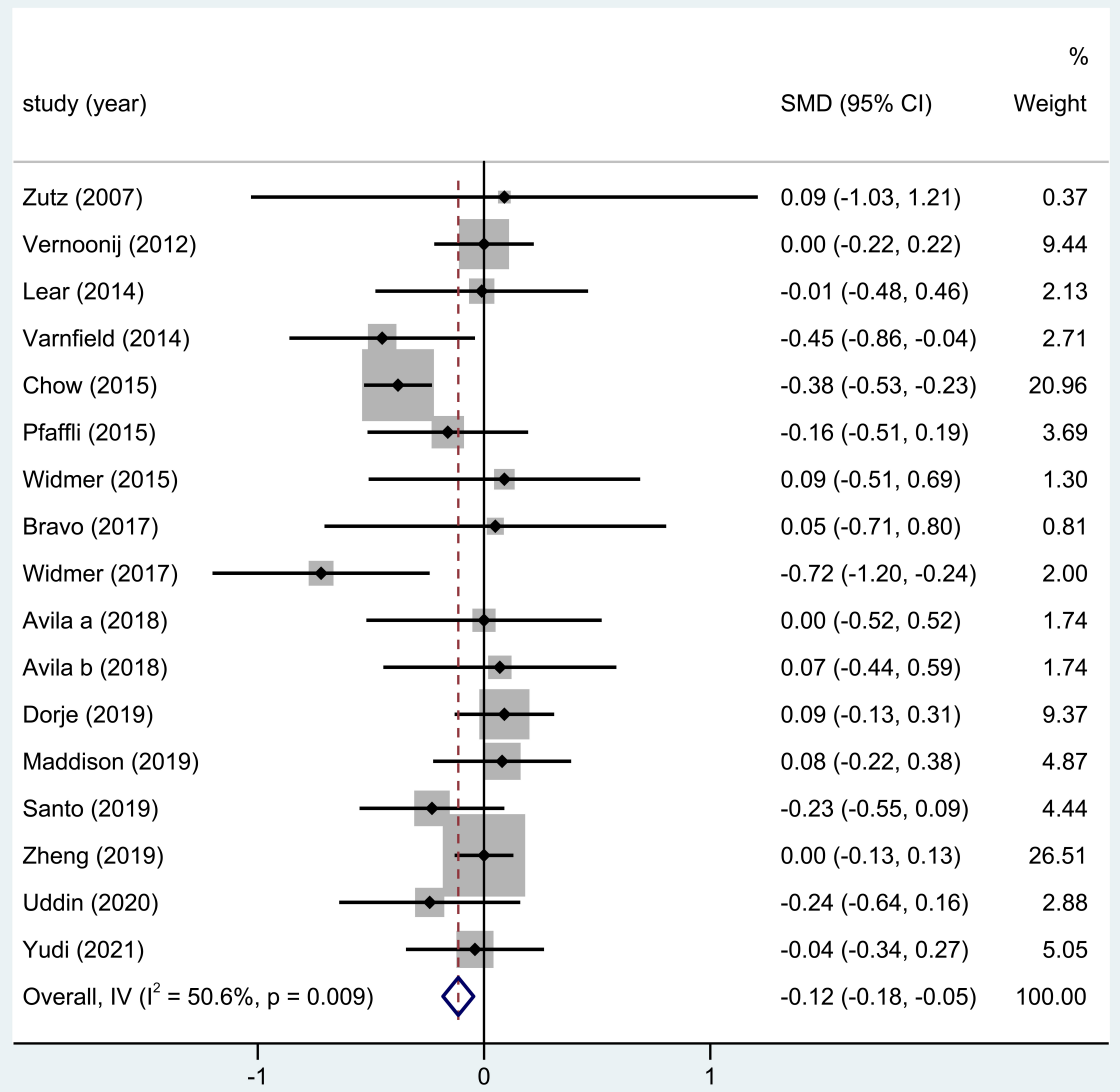
**The effect of home CR treatment on BMI**. CR, cardiac rehabilitation; BMI, body mass index; SMD, standardized mean difference; CI, confidence interval.

Sixteen trials reported on the BMI, and the outcomes of the whole study exhibited obvious differences in the BMI of patients after home-based CR (SMD = –0.12; 95% CI: [–0.18, –0.05], Fig. 8).

### 3.4 Subgroup Analysis

The following parameters were used to perform subgroup analysis: sample size (<100 or ≥100), publishing year, and area. However, no potential causes of heterogeneity were identified in our investigation (**Supplementary Tables 1–7**).

### 3.5 Study Quality 

No evidence of publication bias was observed in the findings for TG, TC, HDL, LDL, SBP, DBP, and BMI, according to Egger’s test and Begg’s funnel plot; more information is provided in **Supplementary Table 8 **and** Supplementary Fig. 1**, and most studies presented with a low risk of bias. Participants were not blinded in four of the studies [12, 17, 20, 27], the results of which are shown in **Supplementary Figs. 2** and **3**.

## 4. Discussion

This study focused on the effectiveness of technology-based home CR in controlling risk factors in patients with CHD. We collected all the technology-related delivery methods (eHealth is the practice and delivery of healthcare using information and communication technologies (ICTs), such as the internet and mobile devices [30]. Telehealth, which incorporates teleconferencing, smartphone applications, and encrypted communication with access from remote access, is frequently used interchangeably with telemedicine [31]. mHealth is the utilization of mobile devices for medical and public health practices, including cellphones, patient monitoring equipment, personal digital assistants (PDAs), and other wireless appliances of CR, and was used to evaluate their effect on reducing risk factors, such as lipid profiles and BMI, SBP, and DBP). According to our findings, technology-based home CR can drastically reduce blood LDL, TC, and TG levels, while increasing serum HDL levels. Meanwhile, it could considerably reduce the BMI, SBP, and DBP indices. Our results are consistent with those of a previous study that researched the efficacy of mHealth CR in alleviating risk factors [32]. However, compared with their study, we did not analyze non-English publications but their study did. We restricted the published languages since the English-language articles were of greater quality and more thorough information. Additionally, they included only mHealth CR in their research, while we used all three approaches. Chong *et al*. [9], analyzed the effectiveness of technology-assisted CR in patients with CHD, and their results depicted no significant difference between technology-assisted CR and traditional/center-based CR on modifiable cardiovascular risk variables, such as S/DBP and TC. The differences in our findings could be attributed to the different number of studies included in the analysis. In their meta-analysis, only five papers were included, whereas we included more than 15 investigations. The number of included studies may also have an impact on the presented outcomes. Furthermore, their analysis did not include enough risk factors, based on their results, whereas we also analyzed other crucial variables, such as TG, HDL, and LDL, which are important to the prognosis of CHD. Regarding the high heterogeneity of our results, we conducted a subgroup analysis. Unfortunately, we did not observe any source of heterogeneity in our analysis (**Supplementary Tables 1–7**). This study thoroughly explored the effects of technology-based home CR on the alleviation of risk factors in patients with CHD. In this study, technology-based home CR included eHealth, mHealth, and telehealth services. This study was comprised of only RCTs. Compared with a previous study, we included more approaches related to technology and analyzed more risk factors that might have an impact on the prognosis of CHD.

CR has been shown to have a considerable impact on both the prevalence and death rates related to CHD, as well as the overall quality of life experienced by patients. CR training has been shown to enhance blood circulation and myocardial capability, facilitate CR, and decrease the chance of impairment and mortality [33]. As artificial intelligence (AI) continues to develop, technology-based CR will progress significantly in personalizing care, improving monitoring, and providing support. Due to the AI algorithms, the rehabilitation device can assess a comprehensive range of patient data, encompassing medical history, vital signs, and exercise capability. This analysis facilitates the creation of CR plans that are customized for individual patients and exhibit a high degree of personalization. Furthermore, these plans possess the ability to adapt and evolve over time, in response to the patient’s progress and changing demands. With the assistance of AI, well-designed programs can be combined with patient education, ongoing monitoring, and ongoing support, meaning that technology-based home CR could be successful over the long term.

### Limitations

Our research has several limitations. First, the source of heterogeneity was not found despite using a fixed model for the subgroup analysis. Second, patients in more than 20 studies were predominantly male, and a sex subgroup analysis was not undertaken because there was insufficient data quantifying the CR benefits by gender. Third, only studies written in English were considered in the inclusion criteria for choosing publications, meaning research written in other languages was excluded. Given these constraints, our findings should be cautiously interpreted.

## 5. Conclusions

In summary, this study found that technology-based home CR can lower TC, TG, and LDL levels as well as increase HDL levels and decrease BMI, SBP, and DBP indexes. Considering the efficacy of CR in controlling risk factors, which play a crucial role in slowing disease progression and reducing recurrence and consequences, technology-based home CR may have value in being extensively promoted for patients with CHD.

## Data Availability

All data are available in the manuscript, and they are exhibited in figures and tables.

## Supplementary Material

Supplementary material associated with this article can be found, in the online version, at https://doi.org/10.31083/j.rcm2502059.
