# Supplementary material for: The Effect of Technology-Based Home Cardiac Rehabilitation on Risk Factor Modifications in Coronary Heart Disease Patients. A Systematic Review and Meta-Analysis
Source: Rev Cardiovasc Med. 2024 Feb 5;25(2):59. doi: 10.31083/j.rcm2502059 (PMC11263160; doi:10.31083/j.rcm2502059)
Supplement: Supplementary file 1 [file 2153-8174-25-2-059-s1.zip › 2153-8174-25-2-059-s1/RCM19582-Supplementary Material-V2 for reviewing.pdf]

Reviews in
Cardiovascular Medicine

Supplementary Material

Supplementary Table 1. Subgroup analysis of BMI in heterogeneity

Category / Year	No. of studies	Heterogeneity statistic	*P*	I^2
1 (2000-2010)	1	0.00	-	-
2 (2010-)	16	32.33	0.00	53.6%
**Region**				
0 (US and Canada)	5	6.27	0.18	36.2%
1 (Europe)	4	0.07	0.995	0.0%
2 (Asia-Pacific)	8	23.25	0.002	69.9%
**Sample size**				
0 (<100)	9	9.79	0.28	18.3%
1 (⩾100)	8	21.72	0.03	67.8%

Supplementary Table 2. Subgroup analysis of SBP in heterogeneity

Category / Year	No. of studies	Heterogeneity statistic	*P*	I^2
1 (2000-2010)	1	0.00	-	-
2 (2010-)	17	92.45	0.00	82.7%
**Region**				
0 (US and Canada)	5	2.45	0.65	0.0%
1 (Europe)	4	2.59	0.46	0.0%
2 (Asia-Pacific)	9	79.84	0.00	90.0%
**Sample size**				
0 (<100)	8	3.53	0.83	0.0%
1 (⩾100)	10	83.91	0.00	89.3%

Supplementary Table 3. Subgroup analysis of DBP in heterogeneity

Category / Year	No. of studies	Heterogeneity statistic	*P*	I^2
1 (2000-2010)	1	0.00	-	-
2 (2010-)	15	49.16	0.00	71.5%
**Region**				
0 (US and Canada)	5	5.14	0.27	22.2%
1 (Europe)	4	1.99	0.57	0.0 %
2 (Asia-Pacific)	7	19.24	0.00	68.8%
**Sample size**				
0 (<100)	8	9.65	0.21	27.4 %

1 (⩾100)	8	22.28	0.00	68.6%

Supplementary Table 4. Subgroup analysis of total cholesterol in heterogeneity

Subgroup	No. of studies	Heterogeneity statistic	*P*	I^2
**Year**				
1 (2000-2010)	1	0.00	-	-
2 (2010-)	13	64.51	0.00	82.7%
**Region**				
0 (US and Canada)	5	30.89	0.00	87.0%
1 (Europe)	1	0.00	0.00	81.8%
2 (Asia-Pacific)	8	38.54	0.00	81.3%
**Sample size**				
0 (<100)	6	17.64	0.00	71.6%
1 (⩾100)	8	43.96	0.00	84.1%

Supplementary Table 5. Subgroup analysis of HDL in heterogeneity

Subgroup	No. of studies	Heterogeneity statistic	*P*	I^2
**Year**				
1 (2000-2010)	1	0.00	-	-
2 (2010-)	12	66.64	0.00	83.5%
**Region**				
0 (US and Canada)	5	8.05	0.09	50.3%
1 (Europe)	2	19.08	0.00	94.8%
2 (Asia-Pacific)	6	38.28	0.00	86.9%
**Sample size**				
0 (<100)	6	23.71	0.00	78.9%
1 (⩾100)	7	42.67	0.00	85.9%

Supplementary Table 6. Subgroup analysis of LDL in heterogeneity

Subgroup	No. of studies	Heterogeneity statistic	*P*	I^2
**Year**				
1 (2000-2010)	1	0.00	-	-
2 (2010-)	14	66.64	0.00	83.3%
**Region**				
0 (US and Canada)	5	23.68	0.09	83.1%
1 (Europe)	2	20.14	0.00	95.0%
2 (Asia-Pacific)	8	38.28	0.00	79.4%
**Sample size**				
0 (<100)	8	43.36	0.00	83.9%
1 (⩾100)	7	28.48	0.00	78.9%

Supplementary Table 7. Subgroup analysis of Triglycerides in heterogeneity

Subgroup	No. of studies	Heterogeneity statistic	*P*	I^2
**Year**				
1 (2000-2010)	1	0.00	-	-
2 (2010-)	10	55.87	0.00	83.9%

**Region**					
0 (US and Canada)	5	12.61		0.09	68.3%
1 (Europe)	1		0.00	0.00	-
2 (Asia-Pacific)	5	32.23		0.00	87.6%
**Sample size**					
0 (<100)	5	9.08		0.06	56.0%
1 (⩾100)	6	38.02		0.00	86.8%

Supplementary Table 8. Metabias of all studies

	Begg's p	Egger's p (95% CI)
Total Cholesterol	0.38	0.12 (-0.70, 5.17)
Triglycerides	0.94	0.22 (-1.45, 5.53)
HDL	0.16	0.78 (-3.64, 2.80)
LDL	0.77	0.34 (-1.53, 4.19)
BMI	3.03	0.91 (-1.46, 1.62)
SBP	0.76	0.06 (-0.07, 4.31)
DBP	0.05	0.01 (0.59, 4.22)

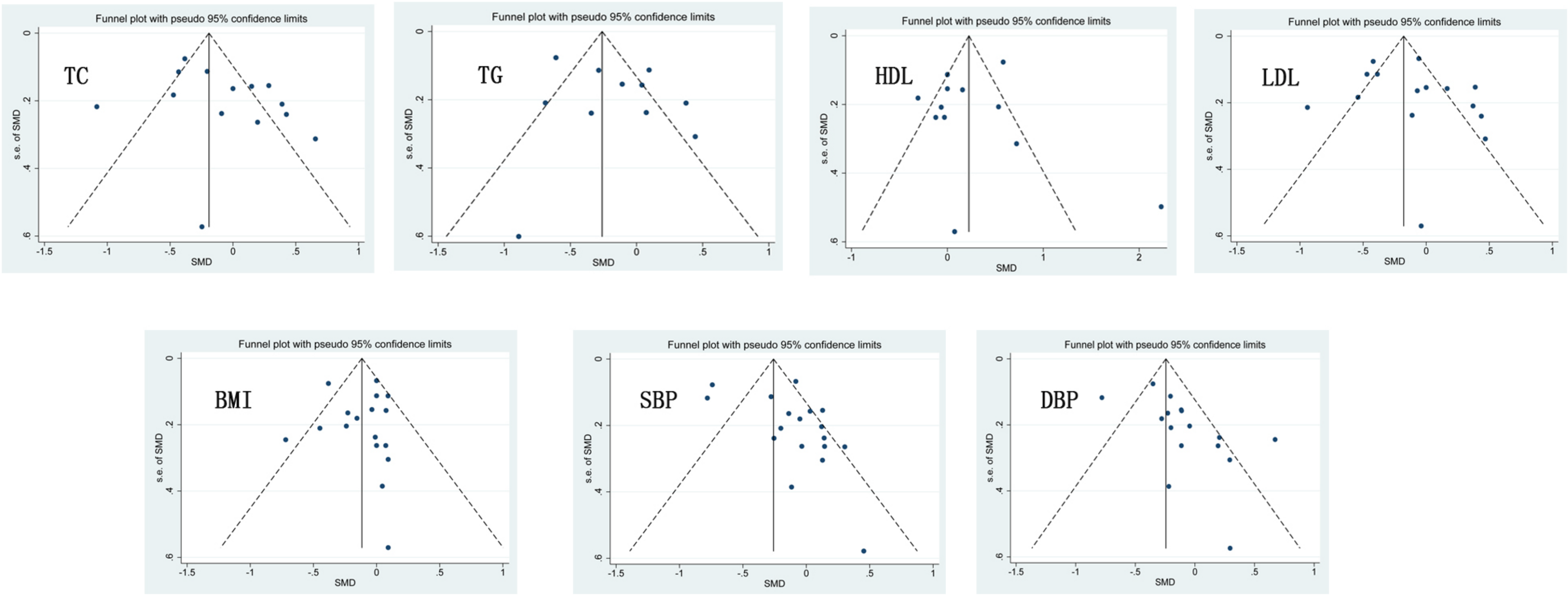

Supplementary Fig. 1. Metafunnel of TC, HDL, LDL, TG, SBP, DBP, BMI. TC: total cholesterol; HDL: high-density lipoprotein; LDL: low-density lipoprotein; TG: triglyceride; SBP: Systolic blood pressure; DBP: Diastolic blood pressure; BMI: body mass index

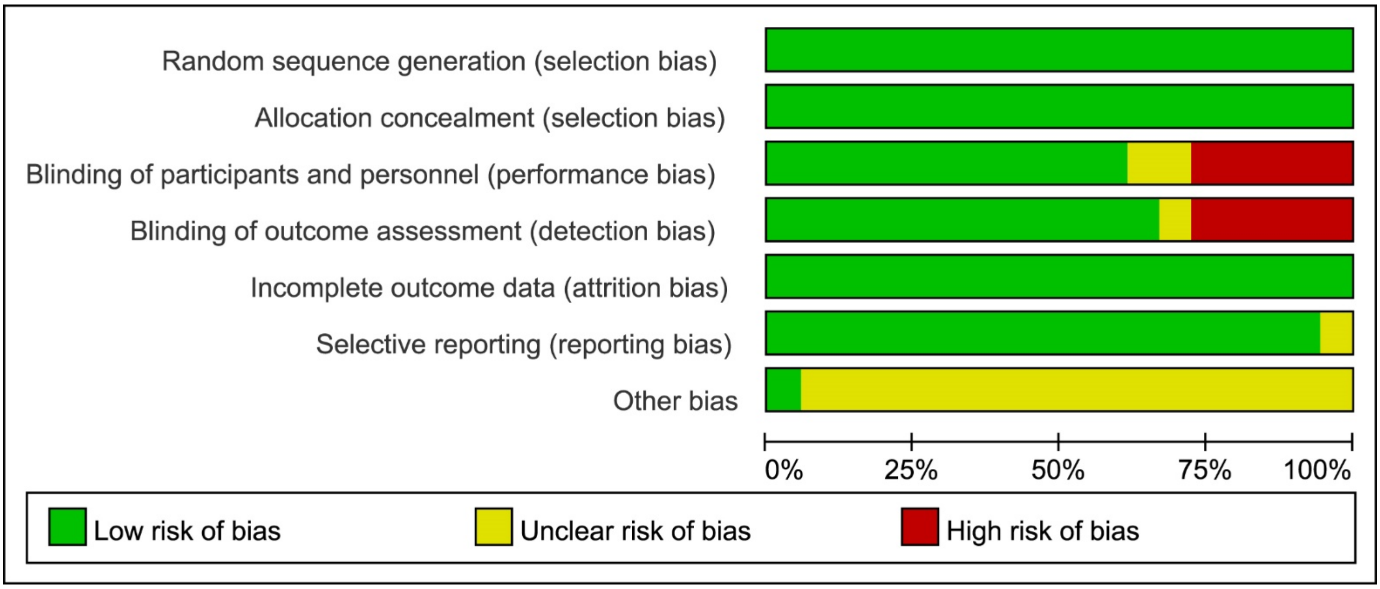

Supplementary Fig. 2. Risk of bias graph

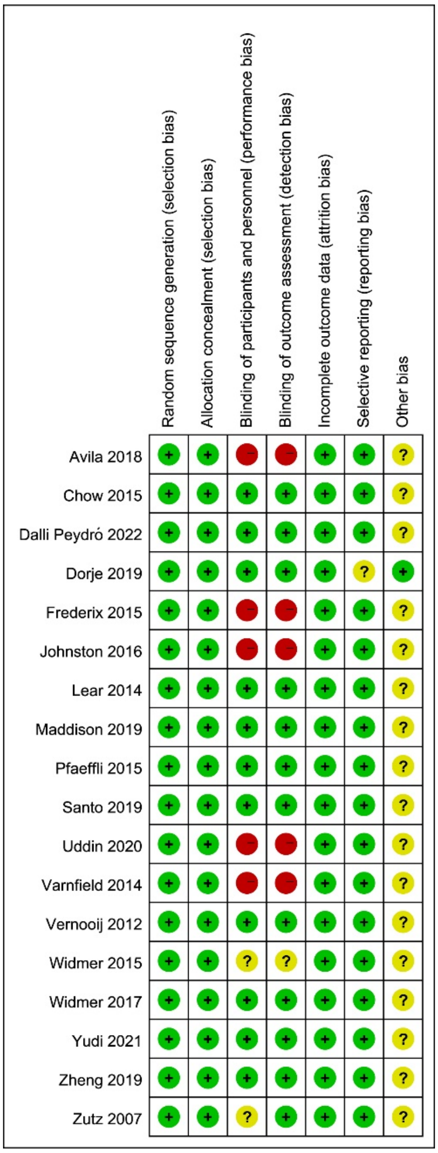

Supplementary Fig. 3. Risk of bias summary
